# Study of Characteristics of Pregnant Women Who Are Refugees and Aged Under 18 Years

**DOI:** 10.7759/cureus.39169

**Published:** 2023-05-18

**Authors:** Aslihan Ergul, Bahar Yuksel Ozgor

**Affiliations:** 1 Obstetrics and Gynecology, Istinye University, Istanbul, TUR; 2 Obstetrics and Gynecology, Esenler Maternity and Children's Hospital, Istanbul, TUR

**Keywords:** pregnancy under 18, refugee, income, education level, birth rate

## Abstract

Introduction

In recent years, the refugee crisis has become one of the most serious problems. It is well-known that women, individuals aged under 18 years, and pregnant refugees are particularly vulnerable to adverse conditions. In this study, we aimed to determine the characteristics of pregnant refugee women aged under 18 years.

Methodology

Data for pregnant women were recorded prospectively from 2019 to 2021, and pregnant refugee women aged ≤18 years were enrolled in the study. Sociodemographic characteristics of women, gravidity and parity status, the status of regular antenatal care visits and antenatal care visit any time until birth, type of birth, causes for cesarean section, presence of maternal comorbidities, obstetric complications, and baby-related characteristics were recorded.

Results

A total of 134 pregnant refugees were enrolled in the study. A total of 31 (23.1%) women had completed primary school, and two (1.5%) women had completed middle school or high school. Additionally, only 3.7% of women had a regular job, and 64.2% of the refugees had a total family income below the minimum wage. Also, 10.4% of women lived with more than three people outside the nuclear family. Gravidity numbers were one for 65 (48.5%) women, two for 50 (37.3%) women, and more than two for 19 (14.2%) women, respectively. The proportion of women with regular antenatal care visits was 19.4% (26), and 45.5% (61) had irregular antenatal care visits. Anemia and urinary tract infections were detected in 52 (28.8%) patients and seven (5.2%) patients, respectively. The proportion of preterm delivery was 8.9%, and 10.5% of infants had low birth weight. A total of 16 (11.9%) babies required neonatal intensive care unit support.

Conclusions

The present study demonstrated that pregnant women who are refugees and aged under 18 years have low education levels, insufficient family income, and often live in crowded families and even as the second wife of a husband. Moreover, although the birth rate was high in pregnant refugees, the rate of regular antenatal follow-up was low. Finally, this study showed that maternal anemia, preterm birth, and low birth weight were common in pregnant refugees.

## Introduction

The refugee crisis has become one of the most serious problems in recent years. According to the United Nations, almost 90 million people were displaced from their land, and almost 30 million became refugees due to the lack of human rights, economic crisis, climate change, violence, and wars [1]. Previous reports demonstrated that refugees face challenging issues including economic problems, racism, inequality of social rights, housing problems, and difficulty accessing healthcare systems [2]. Also, it is well known that refugees who are women, individuals aged under 18 years, and pregnant are more vulnerable to unfavorable conditions [3].

Previous studies found a decrease in female marriage age and an increase in pregnancy rates among refugees [4-6]. Turkay et al. analyzed the characteristics of pregnant women refugees and stated that pregnant refugees were significantly younger than local pregnant women [4]. In another study, Alnuaimi et al. compared the pregnancy outcomes of refugee and local women, and the anemia rate, cesarean section rate, and proportion of neonates with lower weight were significantly higher among refugees [5]. Moreover, Zhang et al. investigated contraceptive methods in refugee and local women and found that contraceptive methods were used less frequently by refugees [6]. Also, Zhang et al. claimed that insufficient and incorrect information about contraceptive procedures, and superstitions have an important role in refugee refusal of birth control methods [6].

 Although previous studies investigated the characteristics of pregnant refugees, none of these studies focused on pregnancies of refugee women aged under 18 years. In this study, we determined the characteristics of pregnant refugees aged under 18 years.

## Materials and methods

In this study, data on pregnant women were recorded prospectively from April 2019 to May 2021. State sources claim that almost two million refugees live in the region where the study was performed. However, independent sources emphasize that this number is close to six million in reality. Pregnant refugee women aged ≤18 years were enrolled in the study. Approval was obtained from the ethics committee, and informed consent was obtained from all patients' legal representative or their families with the help of a professional translator. Detailed medical history was taken, and a physical examination was performed for all patients. Patients older than 18 years, foreign patients who were not refugees, patients who did not want to participate in the study, and patients with missing data were excluded from the study.

Sociodemographic characteristics, including age, first menstruation age, marriage age, body mass index (BMI), nationality of refugee, educational status, working status, the total income of their family, number of people living together outside the nuclear family, and presence of another wife of the husband, were recorded. Also, gravidity and parity status, the status of regular antenatal care visits and antenatal care visits any time until birth, type of birth, causes for cesarean section, presence of maternal comorbidities, and obstetric complications were noted for each refugee. Finally, baby-related characteristics, including sex and gestational age of baby, birth weight, Apgar score at the first minute and the fifth minute, and neonatal intensive care unit (NICU) requirements after birth were analyzed.

Statistical analysis

Statistical analysis was done with Excel (Microsoft Corporation, Redmond, WA, USA). Quantitative data are given as mean ± standard deviation values. Categorical data are given as *n* (%).

## Results

In the study period, 134 pregnant refugees aged ≤18 years were enrolled in the study. The mean age of pregnant refugees and age at first menstruation was 16.0 and 12.4 years, respectively. The mean BMI of the study population was 25.1 ± 4.6 kg/m^2^. The most common nationalities were Syrian (79.1%) and Afghan (8.2%), and 75.4% of women were not educated. Only, 31 (23.1%) women finished primary school, and two (1.5%) women finished middle school or high school. Additionally, only 3.7% (5) of women had a regular job, and 64.2% of the refugees had a total family income below minimum wage. Also, 10.4% of women lived with more than three people outside the nuclear family, and 16% of women had husbands with another wife in the same house (Table 1).

**Table 1:** Sociodemographic data for patients. *Mean ± standard deviation.

Sociodemographic characteristics (*N *= 134)	
Age (years)^*^	16.0 ± 1.5
Age at first menstruation (years)^*^	12.4 ± 1.7
Marriage age (years)^*^	15.4 ± 1.7
Body mass index (kg/m^2^)^*^	25.1 ± 4.6
Nationality, *n* (%)	
Syrian	106 (79.1)
Afghan	11 (8.2)
Iraqi	4 (3)
Central Asian	6 (4.5)
Other	7 (5.2)
Education, *n* (%)	
None	101 (75.4)
Primary school	31 (23.1)
High school	2 (1.5)
Working status, *n* (%)	
No	129 (96.3)
Yes	5 (3.7)
Total income of the family, *n* (%)	
<6,000 Turkish lira	86 (64.2)
6,000-15,000 Turkish lira	42 (31.3)
>15,000 Turkish lira	6 (4.5)
Presence of health insurance, *n* (%)	90 (67.2)
Number of people living together outside the nuclear family, *n* (%)	
0	62 (46.3)
1	36 (26.9)
2	22 (16.4)
3+	14 (10.4)
Another wife of the husband in same the house, *n* (%)	
No	104 (77.6)
1	22 (16.4)
2	8 (6)

Gravidity numbers were one for 65 (48.5%) women, two for 50 (37.3%) women, and more than two for 19 (14.2%) women, respectively. Also, 64.9% (87) of pregnant refugees had one child and 29.1% (39 women) of pregnant refugees had two children. The proportion of women with regular antenatal care visits was 19.4% (26), and 45.5% (61) had irregular antenatal care visits. Cesarean section was performed in 47 (35.1%) women, and cesarean section history and breech birth were the two most common reasons (28 and 9 women, respectively). Anemia and urinary tract infections were detected in 52 (28.8%) and 7 (5.2%) patients, respectively. Blood transfusion was required in 2.2% (3) of women, and surgical infection was observed in 1.5% (2) of women (Table 2).

**Table 2:** Birth characteristics data.

Birth characteristics (*N* = 134)	
Gravidity, *n* (%)	
1	65 (48.5)
2	50 (37.3)
3+	19 (14.2)
Parity, *n* (%)	
1	87 (64.9)
2	39 (29.1)
3+	8 (6)
Regular antenatal care visits, *n* (%)	26 (19.4)
Antenatal care visit at any time until birth, *n* (%)	
Yes	61 (45.5)
No	73 (54.5)
Type of birth, *n* (%)	
Vaginal	87 (64.9)
Cesarean section	47 (35.1)
Reasons for cesarean section, *n* (%)	
Previous cesarean section	28 (20.9)
Breech birth	9 (6.7)
Maternal factors	5 (3.7)
Fetal factors	5 (3.7)
Maternal health problems, *n* (%)	
None	66 (49.2)
Anemia	52 (28.8)
Diabetes mellitus	3 (2.2)
Hypertension	2 (1.5)
Urinary tract infection	7 (5.2)
Other	4 (3)
Obstetric complications, *n* (%)	
Blood transfusion	3 (2.2)
Infection	2 (1.5)
Atony	1 (0.7)

The proportion of preterm delivery was 8.9%, and 10.5% of infants had low birth weight. A total of 16 (11.9%) babies required NICU support. The Apgar score of babies at the first minute and the fifth minute and the characteristics of the infant are summarized in Table 3.

**Table 3:** Neonate characteristics. NICU, neonatal intensive care unit

Neonate characteristics (*N* = 134)	
Sex, *n* (%)	
Male	69 (51.5)
Female	65 (48.5)
Gestational age, *n* (%)	
Preterm	12 (8.9)
Term	119 (88.9)
Postterm	3 (2.2)
Birth weight, *n* (%)	
<2.5 kg	14 (10.5)
2.5-4 kg	114 (85.1)
>4 kg	6 (4.4)
Apgar score at the first minute, *n* (%)	
0-3	8 (6)
4-6	22 (16.4)
7-10	104 (77.6)
Apgar score at the fifth minute, *n* (%)	
0-3	1 (0.7)
4-6	6 (4.4)
7-10	127 (94.9)
NICU admission after birth, *n* (%)	16 (11.9)

## Discussion

Refugee-related health problems not only put pressure on health systems but also cause serious morbidity and mortality in patients. In addition to the fact that pregnancies under the age of 18 are more risky, the difficulty of accessing the health system in cases such as refugees may adversely affect the health of the mother and baby. Thus, we conducted this study to reveal the characteristics of pregnant refugees aged under 18 years. We found that pregnant refugees had low education levels, insufficient family income, and often lived in crowded families and even as the second wife of their husbands. In addition, although the birth rate was high in pregnant refugees, the rate of regular antenatal follow-up was low. Moreover, maternal anemia, preterm birth, and low birth weight were common in pregnant refugee women.

Previous reports stated the difficulties faced by refugees in continuing their education. Correa-Velez et al. analyzed the educational status of refugees in Australia and stated that two out of five students drop out without completing basic education [7]. In another study, Cankaya and Şimşek evaluated the educational status of women and the impact of educational status on hopelessness level and found that 32.9% of participants finished high school [8]. Additionally, Cankaya and Şimşek demonstrated a correlation between lower educational status and higher hopelessness levels [8]. In this study, the illiteracy rate and the rate of graduating only from primary school was 75%. We think that the reason for this high rate is that the study group included women aged only <18 years. In studies examining older women, they may have received education before becoming refugees. In addition, getting married, being pregnant, or adolescents aged under 18 years having children may also result in the interruption of their education.

Refugees often do not have a regular income or private living space. Jonassen et al. analyzed the working status of refugees in Gaza and stated that only two out of five people had regular work. Also, Jonassen et al. found a significant correlation between lower economic status and the presence of chronic diseases [9]. In another study by Lommel and Chen, they investigated the economic status of refugee families in the US. The authors found that almost half of the refugees did not find a job according to their educational level [10]. Also, according to the study by Lommel and Chen, 61% of the refugees stated that their total family income was insufficient to meet their basic needs [10]. In this study, pregnant refugees aged under 18 years were mostly unemployed and lived in crowded and low-income families. Some of these women were also the second wife of their husbands.

Antenatal care visits are crucial for the health of the mother and baby. Masterson et al. analyzed the pregnancies of Syrian refugees in Lebanon and concluded that only 51% of pregnant refugees could access the professional healthcare system [11]. In another study, Kabakian-Khasholian et al. found increases in maternal morbidity and mortality in the absence of antenatal visits in pregnant refugees [12]. According to Huster et al., antenatal visit numbers were significantly higher in Lebanese women than in Syrian refugees [13]. In this study, only 19.4% of all pregnant refugees aged under 18 years attended regular antenatal care. We believe that healthcare institutions should prepare programs emphasizing the importance of antenatal follow-up, and refugees should be provided with more opportunities for antenatal follow-up. Among the most important reasons preventing regular antenatal follow-up are the problems experienced by refugees regarding health insurance.

In addition to the risk of pregnancies aged under 18 years, living in less-than-ideal environments increases the risk of possible complications in pregnancy. Masterson et al. stated that 27% of refugee women experienced anemia during their pregnancy [11]. Although Turkay et al. found hemoglobin levels were significantly higher in locals than in pregnant refugees, the difference was not statistically significant [4]. In this study, anemia was the most common maternal health problem in our study population.

Neonatal health is one of the most worrying issues. Harakow et al. reviewed 19 articles about the health of refugee mothers and babies and found that preterm birth increased for refugees in 16 studies. Also, Harakow et al. stated that the stillbirth risk was increased by 1.2 times in refugees [14]. In another study, Ozel et al. found a 0.6% preterm rate in locals and a 4.7% preterm rate in refugees [15]. In this study, the preterm birth rate for pregnant refugees aged under 18 years was 8.9%. We believe that younger maternal age, lower economic status, and unhygienic living conditions may have led to this situation.

This study has some limitations. This study covered a certain period, and the duration of refugee status among pregnant women was not evaluated. The duration of being a refugee can affect pregnancy-related outcomes, which may be the subject of other studies. Second, the study included information given by patients, and patients may have provided incomplete or misleading information in some cases. Also, in this study, we aimed to reveal the problem but we did not discuss solutions. Solutions to the problems of pregnant refugees aged under 18 years may be the subject of further studies.

## Conclusions

This study demonstrated that pregnant women who are refugees and aged under 18 years have low education levels, insufficient family income, and often live in crowded families and even as the second wife of a husband. Moreover, although the birth rate was high in pregnant refugees, the rate of regular antenatal follow-up was low. Finally, this study showed that maternal anemia, preterm birth, and low birth weight were common in pregnant refugees.
